# Prominent Crystallization Promotion Effect of Montmorillonite on PTT/PC Blends with PTT as the Continuous Phase

**DOI:** 10.3390/polym12030541

**Published:** 2020-03-02

**Authors:** Meiling Xue, Yingjie Liu, Kanghui Lv, Shaowu Han, Shengqiang Gao, Guangshui Yu

**Affiliations:** Key Laboratory of Rubber-Plastics, Ministry of Education/Shandong Provincial Key Laboratory of Rubber-plastics, Qingdao University of Science & Technology, Qingdao 266042, China; liuyingjie_qust@163.com (Y.L.); 15192569335@163.com (K.L.); hanshaowuqust@163.com (S.H.); gaoshengqiang_qust@163.com (S.G.); yugs@qust.edu.cn (G.Y.)

**Keywords:** poly(trimethylene terephthalate), PTT/PC, crystallization, montmorillonite, nucleation

## Abstract

To regulate the crystallization of poly(trimethylene terephthalate) (PTT) retarded by melt blending with polycarbonate (PC), the crystallization of the PTT/PC blend was investigated employing nano-montmorillonite (MMT) as a crystallization promoter with PTT as the continuous phase. The results showed that MMT exhibits a significant promoting effect on PTT crystallization; the presence of 1 wt. % MMT shifts the initial and peak crystallization temperatures of the 70/30 PTT/PC blend to ~17 °C and ~32 °C, respectively. Additionally, the full width at half maximum (FWHM) narrows by ~45%, and the *ΔH_c_* increases by 3.7 J.g^−1^. The accelerating effect of MMT is determined by its distribution and dispersion which depends on the shear intensity, mixing mode, and loading. MMT is easier to exfoliate via the two-step method than by the one-step method. The distribution in the PTT phase is enriched along the phase interface forming an MMT layer. This endows sections of the PTT with abundant nuclei and thus crystallization is promoted markedly compared with the one-step method. Moreover, the finer MMT migrates more readily to the interface to cause a much smoother phase interface. However, a secondary crystallization peak appears when the shear force is not sufficient enough to make MMT finely dispersed, in case of the two-step method and the MMT content is increased to 3 wt. %. The mixing temperature shows little effect on the acceleration of MMT on the crystallization of PTT/PC compared with the shear force. Only when MMT did not exfoliate or uncomplete did the presence of epoxy resin help to promote crystallization because of the improved MMT dispersion.

## 1. Introduction

Poly(trimethylene terephthalate) (PTT) is a semi-crystalline polymer, which has been reported to have outstanding tensile elastic recovery, good chemical resistance, and a relatively low melting temperature [1]. It can be used as a fiber, film, and as an engineering plastic [2,3,4]. As an engineering thermoplastic, it has mechanical properties and favorable processability that are, respectively, comparable to poly(ethylene terephthalate) (PET) and poly(butylene terephthalate) (PBT). Moreover, the smoothness and electrical insulating properties of its products are better than those of PBT and PET. Thus, it possesses some of the advantages of both PET and PBT [1]. However, since it was first synthesized by Whinfield and Dickson in 1941 [5], PTT has not been developed rapidly into a widely used commercial polymer such as PBT and PET have because of the shortage and expensive cost of one of the raw materials,1,3-propanediol (PDO). It was not until 1995 that the Shell Chemical Company announced the industrial production of this material. Following commercial availability in 1998, research concerning PTT gained popularity, especially with regards to its structure and orientation, [2,6,7] crystallization [8,9,10,11,12,13], PTT blends [14,15,16,17,18,19,20], and composites [21,22,23,24,25].

However, the low heat distortion temperature and pronounced brittleness of PTT at low temperature have restricted its use as a desirable engineering plastic. Inspired by PBT/PC blends, which are used as high performance engineering plastics in bumpers, automobile parts, and electrical tools, the development of PTT/PC blends has gained interest because of the excellent properties of PTT and PC; PC possesses pronounced toughness and good heat resistance, while PTT exhibits excellent performance. Such PTT/PC blends can thus be used in automobiles, electrical equipment, outdoor equipment and other fields. Accordingly, a considerable amount of research concerning PTT/PC blends has been reported, with studies relating to blending and compatibility [26,27,28,29,30], phase morphology and rheology [30,31], melting and crystallization [31,32,33], and interfacial reactions [34,35,36,37]. Our group has also reported a series of investigations concerning PTT/PC; for example, we have studied miscibility and compatibilization, phase morphology, rheology, and melting and crystallization behaviors [26,28,30]. However, various reports have shown that the crystallization of PTT is greatly affected by the PC content, leading to the crystallization of PTT/PC being significantly lower than that of PTT [31,33,37]. In order to explore the reasons for the retardation of crystallization when PTT is blended with PC, the relationships between the crystallization behavior and blend composition, as well as the phase morphology, were investigated in detail [38]. The results showed that the predominant reason for the retardation in crystallization is due to the PC content and phase morphology. The PC influences the crystallization of PTT via two methods: firstly, it retards PTT crystallization; secondly, the PC exhibits a nucleation effect on the PTT crystallization; however, this effect is much weaker compared to the negative effect which PC exerts with regards to PTT crystallization.

In addition to PC, the crystallization of PTT has been reported to be restrained by poly(ethylene naphthalate) [39], and perhaps also by other blend components, fillers, reinforcements, etc., when strengthened and modified. This will inevitably influence material properties and rapid production, since the crystallization rate of neat PTT is mild, being an order of magnitude faster than that of PET and an order of magnitude lower than that of PBT [8]. Therefore, the effective regulation of the crystallization of PTT during modification is necessary. Previous studies in our group found that montmorillonite (MMT) exhibits a strong promoting effect on the PTT crystallization, i.e., the presence of 1 wt. % MMT increases the peak crystallization temperature to 22 °C and the enthalpy to 5.4 J.g^−1^, during the non-isothermal crystallization process [40]. Furthermore, the order of promotion of the MMT with different hydrophilic surfaces on PTT crystallization was found to be Closite 30B > Closite 25A > Closite 20A [40]. Based on this, this paper describes the crystallization properties of the PTT/PC blend using MMT as a crystallization accelerator.

Theoretically, the crystallization of PTT/PC in the presence of foreign particles is not only determined by the distribution and dispersion of particles but also by the phase morphology; the phase morphology and the dispersion of particles are in turn influenced by each other. Accordingly, the purpose of the research reported herein is to selectively distribute MMT into the PTT phase preferentially, and to determine the relationship of the crystallization behavior of the PTT/PC blend with the mixing process, the distribution and dispersion of MMT. We aim to effectively control the crystallization of the PTT/PC blends in order to meet various requirements.

## 2. Experimental

### 2.1. Materials

Poly(trimethylene terephthalate), with the trade name Sorona K1171, was supplied by Du Pont China Holding Co., Ltd, Shanghai Branch, China. The polymer had an intrinsic viscosity of 1.02 dL/g. Polycarbonate, LG 1201-15, was purchased from LG Chemical Co., Shanghai, China, with an MFI of 15 g/10 min. The clay used in this study is organically modified montmorillonite, with the trade names of Cloisite^®^ 25A and Cloisite^®^ 30B, as purchased from Southern Clay Products, Inc., Gonzales, TX, USA. The differences in surface treatment agents and surface properties between 30B and 25A are shown in the supporting information, Figure S1. Prior to melt processing, the PTT and PC were dried at 120 °C for 12 h in a vacuum oven to minimize the hydrolytic degradation of the melts, and MMT was dried at 85 °C for 2 h.

### 2.2. Preparation of Blends

The melt blending of the dried PTT, PC, and MMT was carried out using an internal mixer (Haake Rheocord 9000, Thermo Scientific Co., Waltham, MA, USA). The mixing temperature, shear rate and time ranged from 240 to 250 °C, 60 to 100 rpm, and 10 min, respectively. Two mixing methods, one-step and two-step, were used. The one-step method means that PTT, PC, and MMT were put into the mixer together and melt mixed, whereas the two-step method is that PTT was melt mixed with MMT firstly and then added to the PC and mixed continuously. After the mixing was completed, the bulk melt blends were quickly removed from the mixer and pressed into a sheet with thickness of 3–5 mm, then quenched rapidly in ice water to freeze the morphologies of the melt states. Note that all of the above steps were very fast. The samples were then dried at room temperature under vacuum for further measurements by differential scanning calorimetry (DSC), scanning electron microscope (SEM) and transmission electron microscope (TEM). The sample of neat PTT used for the DSC measurements corresponds to the manufacturer provided PTT pellets.

### 2.3. Measurements and Characterization

DSC measurements were carried out using a Netzsch differential scanning calorimeter (DSC-204F1, Netzsch Scientific Instruments, Selb, Germany). The temperature was calibrated with an indium standard. The measurements were performed under a high purity nitrogen atmosphere to minimize the possibility of moisture regain and thermo-oxidative degradation. To avoid uneven thermal conduction through the samples, their weight was maintained at 7.5 ± 0.5 mg. The samples, sealed in aluminum pans, were heated from 0 °C to 270 °C at a heating rate of 10 °C/min and kept at 270 °C for 5 min to eliminate thermal and mechanical history. They were then cooled to 20 °C at a cooling rate of 10 °C/min. The thermograms were recorded during cooling as a function of temperature. The phase morphology was examined by transmission electron microscopy (TEM) and scanning electron microscopy (SEM). (1) SEM sample preparation: The dried blend sheets were freeze-fractured in liquid nitrogen, gold-coated, and then examined using a SEM (JSM-6700F, Japan Electron Optics Laboratory Co., Ltd., Tokyo, Japan); (2) TEM sample preparation: ultrathin sections of the blends were microtomed at room temperature using an Ultratome (Model MT-6000, DuPont Co., Wilmington, DE, USA) equipped with a diamond knife. They were then observed using TEM (JSM-2000EX, Japan Electron Optics Laboratory Co. Ltd., Tokyo, Japan) under an acceleration voltage of 200 kV. Wide angle X-ray diffraction (WAXD) measurements were performed on a Japan Rigaku D/max 2500 m with Cu Kα radiation (λ = 0.154 nm) at room temperature. The sample thickness is 2 mm. The accelerating voltage is 40 kV and a current of 100 mA. Data were collected with a step size of 0.02° from 2θ = 1–10°.

## 3. Results and Discussions

### 3.1. Effect of the Surface Property of MMT on the PTT/PC Crystallization

**Figure 1:**
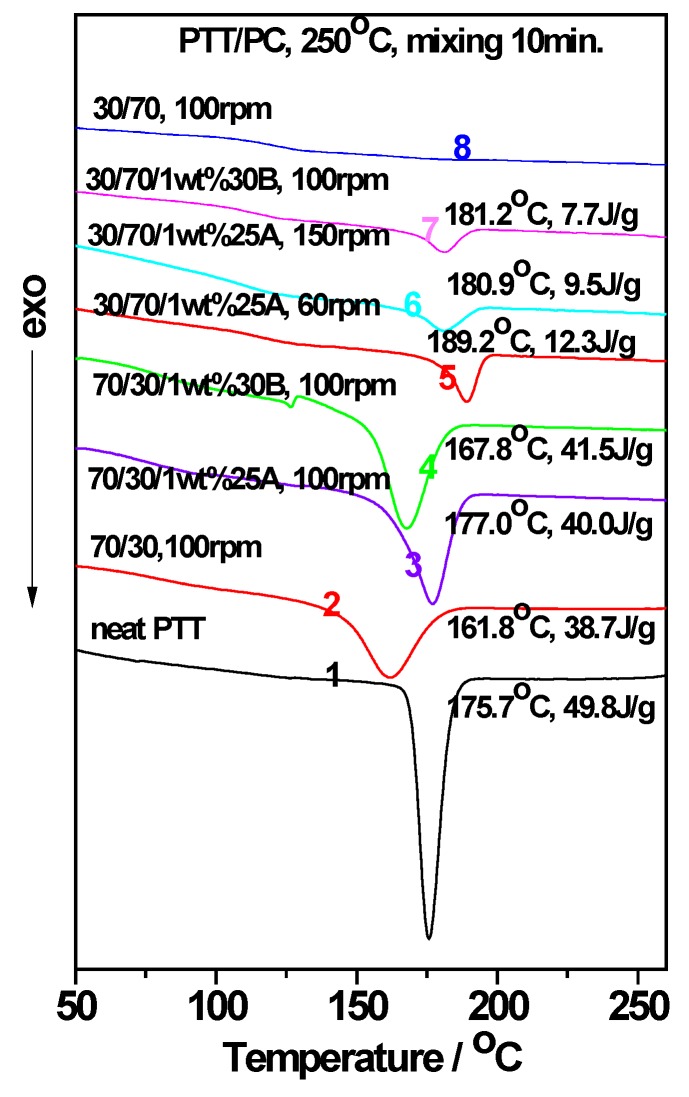
Influence of the surface hydrophobicity of montmorillonite (MMT) (Cloisite^®^ 25A and Cloisite^®^ 30B) on the crystallization behavior of poly(trimethylene terephthalate)/polycarbonate (PTT/PC) blends. The peak crystallization temperature *T_c_* and the apparent crystallization enthalpy *ΔH_c_* are shown.

As mentioned previously, the acceleration effect of Closite 30B on PTT crystallization was more obvious than that of Closite 25A [40]. Moreover, the surface hydrophobicity of 25A was significantly stronger than that of 30B because of the hydroxyl groups on the surface of 30B. 30B and 25A were therefore incorporated into the PTT/PC blend during processing in order to determine their effects on the acceleration of PTT crystallization. The DSC results are shown in Figure 1. The composition, screw rotation, mixing temperature, time, peak crystallization temperature (*T_c_*), and crystallization enthalpy (*ΔH_c_*) of the PTT phase are also indicated. Moreover, to compare the retardation effects of PC and the effects of MMT on the crystallization of the PTT phase, the crystallization behaviors of neat PTT and 70/30 PTT/PC are also provided. It can be seen that the *T_c_* of neat PTT is 175.7 °C. When PTT was blended with PC in a mass ratio of 70/30, the *T_c_* reduces in temperature by 13.9 °C and the full width at half maximum (FWHM) widens by about 130%, showing strong interference with PTT crystallization.

Generally, the intrinsic crystallization ability of polymers depends on their chain structures. During the cooling process after melting, the nucleation rate determines the initial crystallization temperature (*T_i_*), although the growth rate and the nuclei density are also important factors (the nucleation rate and nuclei density, as well as the growth rate, determine the value of *T_c_*). The *ΔH_c_* is related to the nuclei density, the grain size and spherulite perfection. The FWHM of crystallization reflects the multiple levels of the crystallization rates and depends on the rate at which nuclei develop (number of nuclei per time and volume units) and on the growth rate [41]. During processing, both the slow nucleation and low nucleation density can all be artificially accelerated or increased by incorporation of nucleating agents. The intrinsic high growth rate can also be artificially slowed down by using growth inhibitor, but the intrinsic high nucleation cannot. Additionally, the intrinsically low growth rate cannot be increased. Therefore, the changes in crystallization are reflected by the parameter changes of the DSC curves.

As seen from Figure 1, when PTT is the continuous phase, the *T_c_* and *ΔH_c_* of the 70/30 PTT/PC increase due to the presence of 1 wt. % 25A (line 3) or 1 wt. % 30B (line 4); the *T_c_* and *ΔH_c_* in the presence of 1 wt. % 25A are, respectively, 9.2 °C and 1.5 J.g^−1^ higher than the increases observer with 1 wt. % 30B under the same processing conditions. Moreover, the *T_c_* with 1 wt. % 25A (line 3) is higher than that of neat PTT while it is lower than of neat PTT in the presence of 1 wt. % 30B (line 4). However, when PTT is the dispersed phase, both 1 wt. % 25A and 1 wt. % 30B can make 30/70 PTT/PC, which does not crystallize without MMT (line 8), exhibit crystallizations at peak temperatures ranging from 181 to 189 °C (lines 5–7); the crystallization temperatures are much higher than those of neat PTT, exerting strong promoting effects on the crystallization of the PTT dispersed phase. Previous studies showed that the dispersed PTT phase exhibits a fractionated crystallization phenomenon or no crystallization due to the difficulty of homogeneous nucleation [42]. The distinguished accelerating effect of MMT on the PTT dispersed phase reveals that the potential crystallization energy of PTT is promoted. Furthermore, the greater the retardation of the crystallization, the greater the crystallization potential. However, the *ΔH_c_* of the PTT phase in the case of 1 wt. % 25A, whether at 60 rpm (line 5) or 150 rpm (line 6), is obviously higher than that of the corresponding 1 wt. % 30B at 100 rpm (line 7), suggesting that 25A has a stronger ability to induce PTT nucleation. Therefore, 25A was selected as a crystallization promoter for PTT, and taking 70/30 PTT/PC as an example, the effects of the shear rate, mixing mode, temperature, and phase interface on the distribution and dispersion of MMT in the PTT/PC matrix, and thereby on the crystallization of the PTT phase, were further investigated.

### 3.2. The Acceleration of MMT on the Crystallization of PTT/PC: Effect of the Mixing Mode

**Figure 2:**
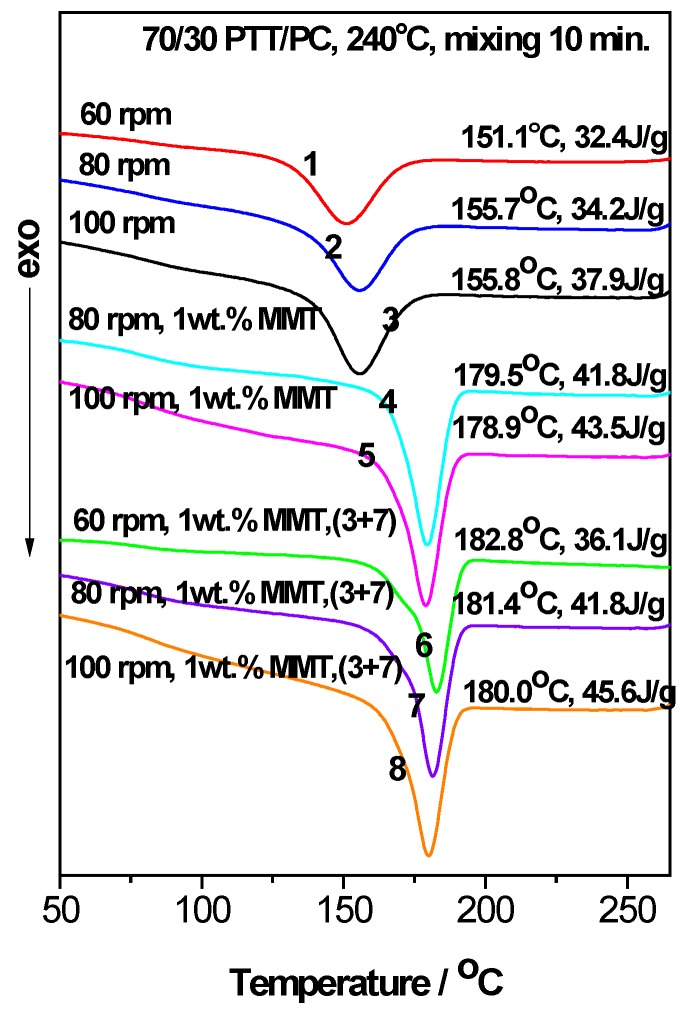
Influence of the shear rate, MMT (Cloisite^®^ 25A) and processing mode on the crystallization of the 70/30 PTT/PC blend processed at 240 °C for 10 min. (3 + 7) signifies that PTT was mixed with MMT for 3 min and then added to the PC and mixed continuously for 7 min.

**Figure 3:**
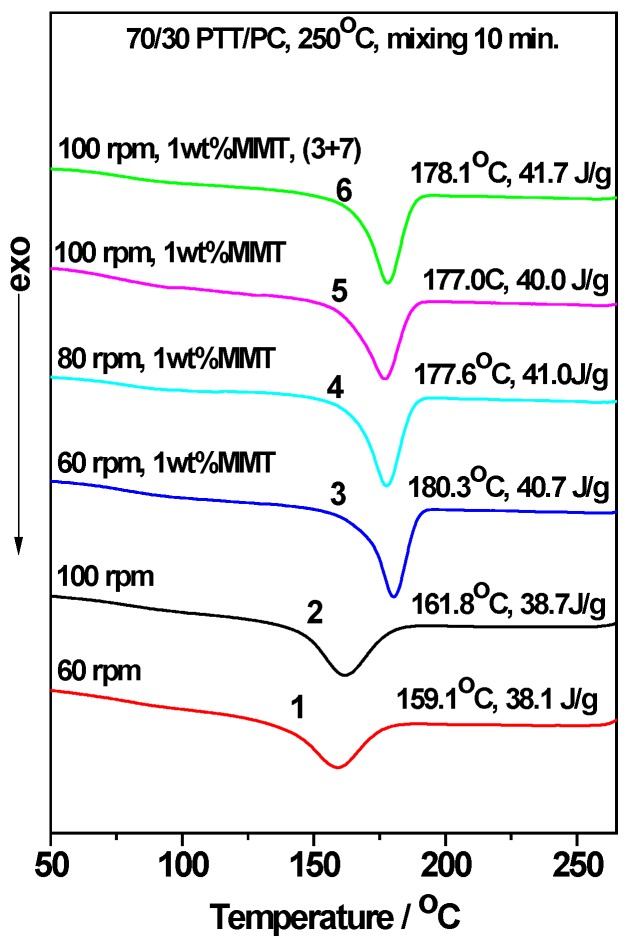
The influence of shear rate, MMT (Cloisite^®^ 25A) and processing mode on the crystallization of the 70/30 PTT/PC blend processed at 250 °C for 10 min. (3 + 7) signifies that PTT was mixed with MMT for 3 min and then added to the PC and mixed continuously for 7 min.

Figure 2 and Figure 3 show the roles of the mixing method and shear rate for the effect of MMT on the crystallization behavior of the 70/30 PTT/PC blend melt mixed at 240 °C and 250 °C, respectively. For convenience, the *T_c_* and apparent *ΔH_c_* values are shown in the upper-right of each line in Figure 2 and Figure 3. It should be noted that the apparent *ΔH_c_*, owing to the mass ratio of PTT to PC, is fixed at 70/30, and can be used to compare the relative crystallinities of the samples. In addition, the (3 + 7) signifies the two-step mode of PTT and MMT was mixed for 3 min, and then PC is added and mixed continuously for another 7 min.

Figure 2 and Figure 3 show that MMT exhibits a prominent ability to promote the crystallization of the PTT phase, evidenced by the initial crystallization temperature (*T_i_*) and the *T_c_* of the PTT phase being shifted to higher temperatures; this shift is accompanied by a marked narrowing of the FWHMs in the presence of MMT. For example, at 240 °C and 60 rpm (line 6 vs. line 1, Figure 2), the presence of 1 wt. % MMT shifts the *T_i_* and *T_c_* values higher by ~17 °C and ~32 °C, respectively. Additionally, the FWHM narrows by ~45%, and the *ΔH_c_* increases by 3.7 J.g^−1^ or ~11% compared with those of MMT absence, showing that nucleation and growth are promoted and that the multi-level crystallization phenomenon caused by PC is significantly weakened.

**Figure 4:**
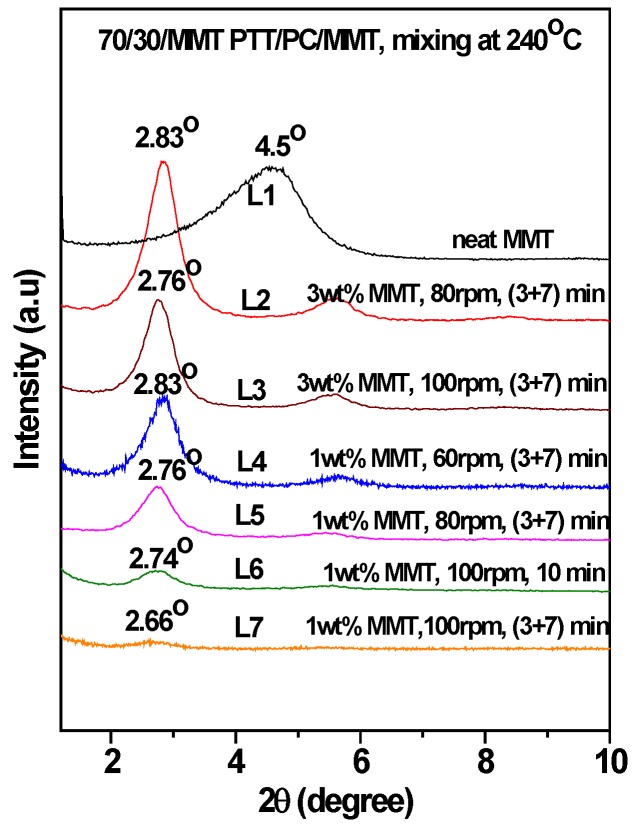
The wide-angle X-ray diffraction (WAXD) patterns of MMT (Cloisite^®^ 25A) in the 70/30 PTT/PC blends processed at 240 °C with indicated shear and processing mode.

**Figure 5:**
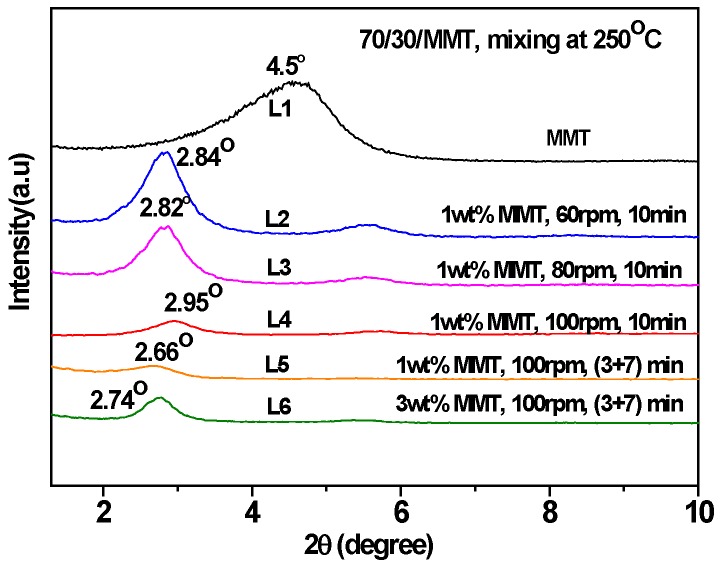
The WAXD patterns of MMT (Cloisite^®^ 25A) in the 70/30 PTT/PC blends processed at 250 °C with indicated shear and processing mode.

**Figure 6:**
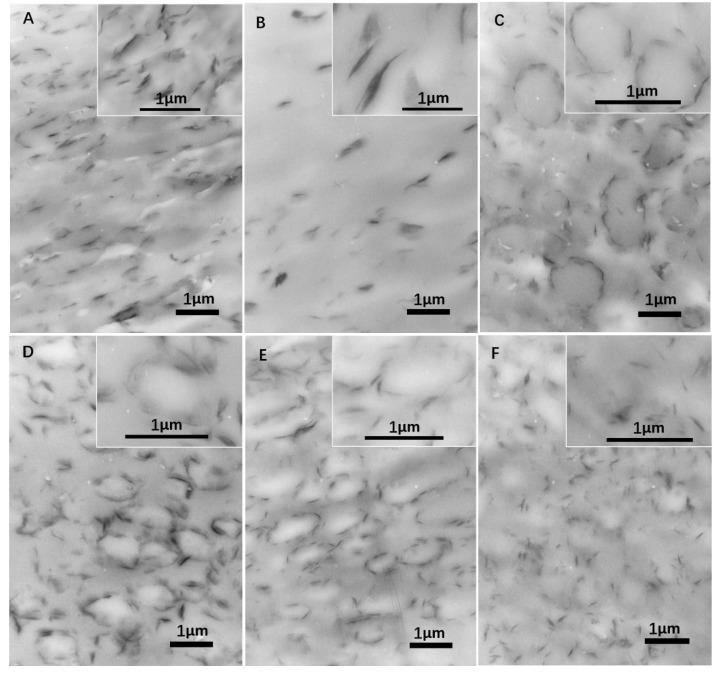
TEM images of the 70/30/MMT PTT/PC blends processed at 240 °C with varied shear rate, processing mode, and MMT (Cloisite^®^ 25A) loading, with or without epoxy resin. (**A)**: 1 wt. % MMT, 60 rpm, (3 + 7min); (**B**): 1 wt. % MMT, 0.5 wt. % epoxy resin, 60 rpm, (3 + 7) min; (**C**): 1 wt. % MMT, 80 rpm, (3 + 7) min; (**D**): 3 wt. % MMT, 80 rpm, (3 + 7) min; E: 1 wt. % MMT, 100 rpm, (3 + 7) min; F: 1 wt. % MMT, 100 rpm, 10 min.

Under the two different shear rates at 240 °C (Figure 2, line 7 vs. line 4, line 8 vs. line 5) and mixing via the two-step method, the *T_c_* and *ΔH_c_* are slightly higher than those of the corresponding one-step method, the same is true for mixing at 250 °C (Figure 3, line 6 vs. line 5). The different promoting abilities of MMT on the crystallization between the two mixing methods reveals that its distribution and dispersion are different. To verify this, WAXD and TEM were employed and the results are shown in Figure 4, Figure 5 and Figure 6. Figure 4 is the WAXD patterns of MMT in the 70/30 PTT/PC blends processed at 240 °C with indicated shear and processing mode. For comparison, the diffraction of neat MMT powder (Cloisite^®^ 25A) is shown as L1, which exhibits a (001) diffraction peak at 2θ = 4.5° and corresponds to an interlayer spacing of 1.96 nm. However, the diffraction peaks shifted markedly to the lower angles in the 70/30/MMT PTT/PC blends, i.e., a peak is shown at 2θ = 2.74° for 70/30/MMT (1 wt. %) when it processed at 100 rpm and via the one-step method (line L6), which corresponds to an interlayer spacing of 3.22 nm. Since the thickness of each sample is 2 mm, the effect of processing conditions on the degree of MMT exfoliation can be compared among the samples with the same MMT loading. Accordingly, the lower intensity suggests that MMT is dispersed in intercalation- exfoliation state but mostly in exfoliation. In contrast, at the same shear rate using the two-step mixing method, the (001) diffraction of MMT almost disappears (line L7), indicating almost complete exfoliation. Figure 5 shows the WAXD patterns of MMT in the 70/30 PTT/PC blends processed at 250 °C. Comparing line L4 with line L5, it can be seen that MMT is exfoliated nearly completely by the two-step method, and the layer spacing of the intercalated sections is also larger than that in the one-step method. Furthermore, the degree of MMT exfoliation in the one-step method is significantly lower than that in the two-step method.

Figure 6 shows the distribution and dispersion of MMT in the PTT/PC matrix. The images show that MMT, when the sample is processed either via the two-step or one-step method at 240 °C and 100 rpm, exists predominantly in an exfoliated state, and the interlayer spacing of the small amount of non-exfoliated MMT increases close to that of the exfoliated state, which is consistent with the results shown in Figure 4. However, dispersion via the one-step method is obviously different from that of the two-step method. In the first case, MMT is basically uniformly dispersed in the matrix, although it is slightly enriched at the phase interface; however, it is mostly distributed either in the PTT phase or along the phase interface in the second case, and preferentially forms an MMT layer in the interface.

Generally, for the two-phase structure, the crystallization interference of one phase with the other occurs at the interface. The interference of the PC phase with crystallization of the PTT phase is an example of this [38]. That is, for the PTT/PC blend, the crystallization ability of PTT in the interface is weak. However, the enrichment of MMT in the interface via the two-step method, by chance, endows precisely this weak section of PTT with abundant nucleation resources, resulting in a marked increase in crystallization. Simultaneously, the decreased crystallization ability of PTT near the interface area, due to interference of the PC, is offset. This is most likely the reason as to why the two-step method is better than the one-step method in inducing nucleation, and also the reason that the nucleation induced by MMT via the two-step method is obviously stronger than the one step process.

### 3.3. The Acceleration of MMT on the Crystallization of PTT/PC: Effect of Shear

In general, the greater the shear rate, the better the dispersion of the nucleating agent in the matrix; the more potential nuclei that are formed, the stronger the crystallization ability. This also occurs in the two-phase structures in the absence of a nucleating agent. In this case, the effect of the shear rate on crystallization is influenced by the phase morphology [38], i.e., the phase domain size, the interface area and the compatibility, etc. However, the effect of the phase morphology on the crystallization (promotion or inhibition) is significantly smaller than that of the nucleating agents. This is why when the shear is increased from 60 to 80 rpm and then 100 rpm, its effect on the PTT crystallization is not significant compared to that of the MMT content (lines 1–3 in Figure 2 and lines 1–2 in Figure 3), i.e., the *T_c_* and *ΔH_c_* of the 70/30 PTT/PC in the presence of 1 wt. % MMT, when processed by the one-step method at 240 °C and 80 rpm, are increased by 28.4 °C and 9.4 J.g^−1^, respectively. This is even greater than the effect of 1 wt. % MMT on neat PTT [40], suggesting that MMT has a stronger nucleating and crystallization promoting effect on the PTT phase. When the rotation is increased to 100 rpm, the *T_c_* shows no obvious change, but the *ΔH_c_* increases by 1.7 J.g^−1^ (Figure 2, line 5), which indicates that the accelerating effect of MMT on the crystallization promotion is enhanced with an increase in shear rate. Based on this, a series of experiments were designed to explore how the shear rate influences the distribution and dispersion of MMT in the PTT and PC phases; the crystallization of the PTT phase was then determined.

**Figure 7:**
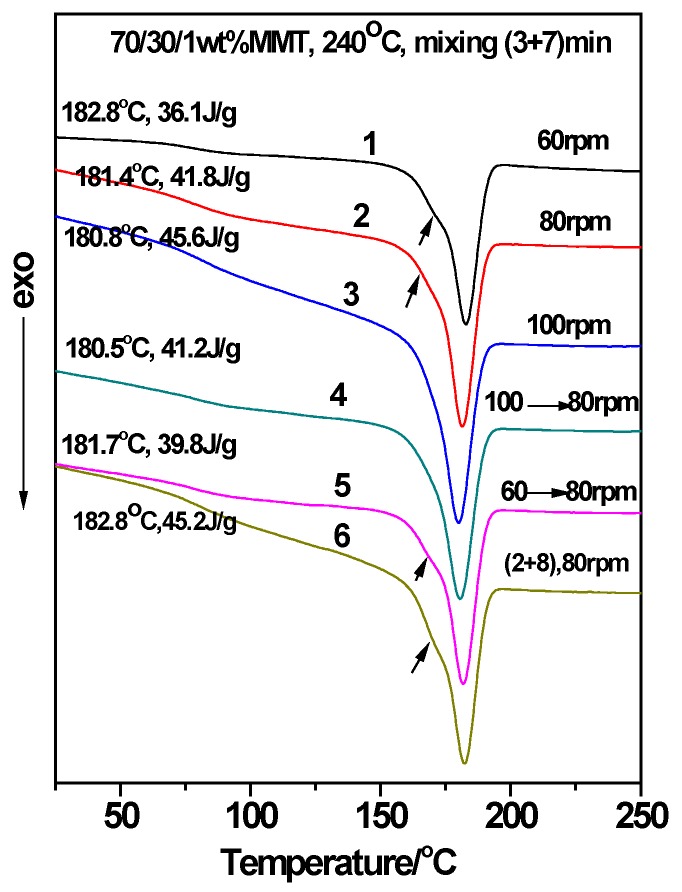
The influence of shear rate on the crystallization of the 70/30/1 wt. % PTT/PC/MMT blend processed at 240 °C for 10 min. The MMT is Cloisite^®^ 25A.

Given 1 wt. % MMT and a two-step mode of (3 + 7), the 70/30 PTT/PC were mixed at 60 rpm, 80 rpm, and 100 rpm for 10 min, respectively, to investigate the effect of shear rate on the crystallization of the PTT phase. From results shown in lines 1–3 in Figure 7, it can be seen that following an increase in the shear rate from 60 to 100 rpm, the *T_c_* decreases slightly by approximately 2 °C, but the *ΔH_c_* increases from 36.1 to 45.6 J.g^−1^. This shows that better dispersion of MMT, promoted by the increased shear rate, is more beneficial to improving nucleation on PTT compared with raising the *T_c_*. WAXD and TEM were employed to provide evidence of this viewpoint. The line L4 in Figure 4, with a shear speed of 60 rpm, shows that the 2θ of the MMT (001) plane shifts from 4.5° in neat MMT to 2.83° in the PTT/PC matrix, indicating that the polymers are intercalated into the MMT layers. The TEM image (Figure 6A) shows that MMT is partially exfoliated and partially intercalated in this case. When the rotation is increased to 80 rpm (line L5), the diffraction angle changes slightly, but the diffraction intensity is obviously weakened, showing that the dispersion of MMT is further improved, and the image C in Figure 6 confirmed this. As the rotation is further increased to 100 rpm (line L7), the diffraction of the (001) plane almost disappears, indicating that the exfoliation of MMT is nearly complete. However, a weak diffraction of the (001) plane at 2θ = 2.74° is still clearly visible in the sample mixed by the one-step method, indicating that there is still a small amount of MMT, which has not been exfoliated and still in intercalated state. The above results show that the greater the shear rate, the higher the ratio of exfoliation of MMT. This results in a higher density of induced nuclei and greater crystallinity of the PTT phase.

Figure 6 shows that MMT, mixed by the two-step method, is mainly enriched along the phase interface at partially exfoliated and intercalated state, forming a spherical MMT layer oriented along the interface. The others are distributed in the PTT phase rather than being uniformly dispersed between the two phases. Moreover, the dispersion of MMT with increased shear rates changes as follows. At 60 rpm, although partially exfoliated and partially intercalated in the PTT phase and interphase area, its enrichment along the interface is still not significant, and is not mainly distributed in the interface, revealing that the shear rate is insufficient to exfoliate MMT at high ratios. This kind of shear deficiency is evidence on the one hand, by not being fast enough to exfoliate the intercalated MMT and, on the other hand, being insufficient to allow MMT to migrate to the interface area within a limited residence time. Comparatively, although MMT is also distributed in a partially exfoliated/intercalated state both in the PTT phase and interphase area at 80 rpm, the exfoliation ratio is obviously raised, and is more inclined to form a spherical MMT interface layer with a diameter of 0.5–1.0 µm distributed along the tangential interfaces. At 100 rpm, MMT is exfoliated almost completely and refined, which is consistent with the WXAD result shown in Figure 4. Similarly, MMT is mainly distributed in the PTT phase and enriched in the interface forming an oriented MMT layer along the phase interface, but the diameter of the MMT layer is significantly reduced to 0.3–0.5 µm, and the phase domain is also obviously reduced. This means that the increased shear rate is beneficial to MMT exfoliation and that the finely dispersed MMT also interferes with the dispersion/coalescence balance of the dispersed phase. This hinders particle aggregation and thus provides a refined phase morphology. Comparatively, MMT is exfoliated almost completely into very fine dimensions in the sample mixed via the one-step method at 100 rpm. However, differing from the two-step method, the MMT is uniformly dispersed in the matrix, rather than distributed mostly along the interface orientation. The above results show that the dispersion of MMT in the matrix depends on the shear intensity, and the two-step method can influence the distribution resulting in enrichment at the interface or distribution into the PTT phase.

In summary, at a low processing temperature such as 240 °C and with a faster shear rate, the better the dispersion of the MMT. This leads to the induction of more nuclei and thus a stronger ability to promote the crystallization of PTT and thus greater crystallinity. At the same rotation, the two-step method helps to improve the dispersion of MMT and allows it to be selectively distributed in the phase interface and the PTT phase, resulting in the formation of a spherical MMT layer oriented along the interface. This joint effect results in MMT providing a strong nucleating ability at the PTT phase, releasing the crystallization potential contained in the PTT phase. This partially overcomes the inhibition of the spherulite growth exerted by the PC segment near the phase interface, thereby making the *T_c_* and *ΔH_c_* values higher than those mixed via the one step method at the same rotation speed.

**Figure 8:**
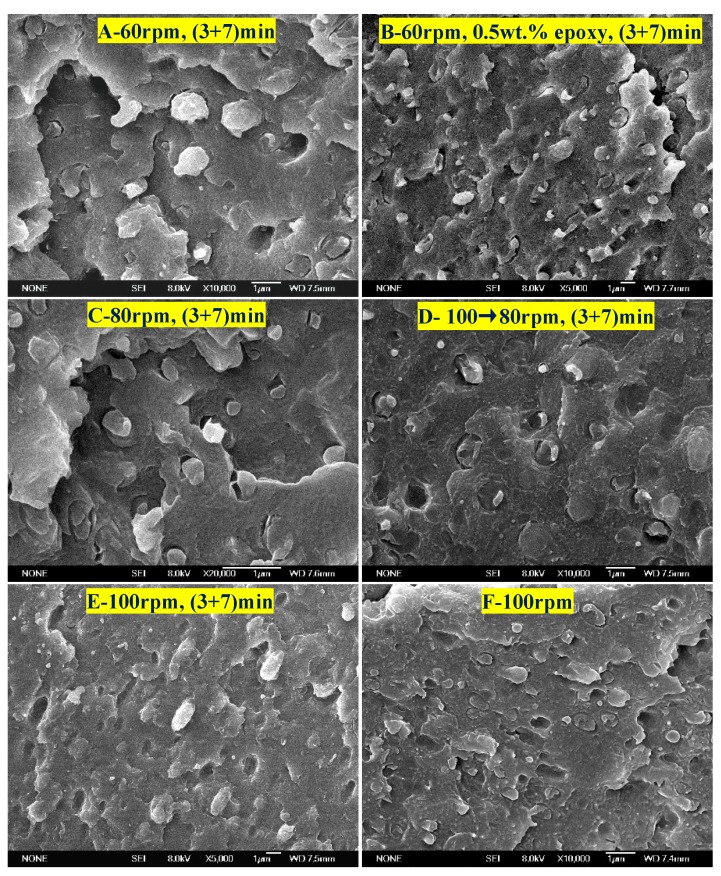
SEM images of the 70/30/1 wt. % PTT/PC/MMT melt processed at 240 °C with the indicated shear rates and by using the two-step mode of (3 + 7) min. The 100→80 rpm denotes that PTT was mixed with MMT at 100 rpm for 3 min firstly and then PC was added and mixing continued for 7 min. Sample D was with 0.5 wt. % epoxy resin addition. The MMT is Cloisite^®^ 25A.

This leads to the question of what effect an increase in shear speed has on the morphology of the PTT/PC in the presence of MMT. Figure 8 provides the answer. It can be seen that PC, as the dispersed phase, exhibits rock-like rough polyhedral spheres with a dimension of 0.5–1 µm at a 60 rpm rotation, owing to the influence of the coarse dispersion of MMT and poor exfoliation; the phase interface is also a rough polyhedral shape. At 80 rpm rotation, the PC phase is refined as a particle size of 0.3–0.4 µm, and the surface roughness is obviously improved. However, at 100 rpm, the phase morphology changes significantly in that the PC phase and the phase interface are all round and the rock-like roughness disappears; the PC phase shows ellipsoids with diameters of 0.5–1 µm. The TEM results in Figure 6 also show that the finely dispersed MMT is distributed along the interface orientation forming a round spherical interface layer, and the PC phase is ellipsoid in shape with round surfaces. The dimensions around the dispersed phase shown by TEM and SEM are consistent.

To further confirm the effect of the shear speed on the dispersion of MMT and the phase morphology, and hence on the crystallization of the PTT phase, various shear and mixing times were investigated during mixing via the two-step method. For example, PTT and MMT were mixed for 3 min at 100 rpm first, PC was added next, and then the rotation immediately reduced to 80 rpm and mixed continuously for 7 min. The DSC result is shown as line 4 in Figure 7. It can be seen that the *T_c_* and *ΔH_c_* values compared with the (3 + 7) mode (line 2) under a constant shear speed of 80 rpm, decrease by 0.9 °C and 0.6 J.g^−1^, respectively, suggesting that the promoting effect of MMT on the crystallization remains mostly unchanged. However, as seen from the morphologies in Figure 8C,D, the dimensions of the dispersed phase for the sample obtained via the variable shear rate process is 0.2–0.8 µm, whereas it is 0.2–0.6 µm for the sample prepared at a constant shear rate. This shows that the dispersion/coalescence equilibrium of the phase domains exerted by MMT is disrupted during the variable shear rate process, owing to the improved dispersion of MMT. This results in a reduction in the ability to block the aggregation of PC, and thus, the PC phase domain remains coarse, reducing the rate of crystallization. On the other hand, the improved dispersion of MMT promotes the nucleation of the PTT phase. The two competing effects results in the *T_c_* and *ΔH_c_* of the PTT phase remaining unchanged. This also provides the reason as to why the particle size shown in Figure 6F is smaller than that in Figure 6E.

The corresponding DSC result for mixing PTT and MMT first at 60 rpm for 3 min, adding PC, increasing the rotation 80 rpm and mixing for a further 7 min, is shown in line 5 in Figure 7. Compared with samples obtained via the mixing mode of (3 + 7) under a constant shear rate of 80 rpm, the *ΔH_c_* decreases by 2 J.g^−1^ whereas the *T_c_* remains unchanged (line 2), which proves once again that the poor dispersion of MMT results in decreased nucleating ability. If the rotation remains at 80 rpm and the (3 + 7) mode is changed to the (2 + 8) mode, that is, the first step time is reduced to 2 min from 3 min (line 6), then the *ΔH_c_* is increased by 3.4 J.g^−1^, and the *T_c_* is increased by 1.4 °C.

In addition, the DSC curves in Figure 7 show that there appears to be secondary crystallization peaks (lines 1, 2, 5, and 6, arrow indicated) which overlap with the main peaks when the rotation is less than 100 rpm, whereas they disappear as the rotation reaches 100 rpm. The reason for this can be seen from the WAXD diffraction patterns and TEM images. One reason is that MMT does not exfoliate at large fractions when the rotation is less than 100 rpm. The other reason is that the potential nucleation resources are distributed along the phase interface in large enough quantities to counteract the interference of the PC segments on PTT crystallization; this results in a difference in the crystallization rate between bulk PTT and the PTT in the phase interface. The appearance of the second crystallization peak is most likely a result of the dispersion of MMT not being pronounced enough, which leads to an obvious difference in the crystallization rates between the inner and the outer layers in the PTT phase.

According to the above results, it is confirmed that: (1) the increased shear rate leads to the improvement in the MMT dispersion and therefore results in an increased nucleation density. This promotes crystallization which is hindered by inhibition of the PC segment, and consequently, the crystallinity of the PTT phase is increased. Moreover, finer MMT preferentially orients along the phase interface, resulting in smoother phase interfaces; (2) the two-step method allows for MMT to be selectively distributed in the phase interface and the PTT phase. A spherical MMT layer forms along the orientation of the interface, which can significantly promote the nucleating ability of MMT resulting in higher *T_c_* and *ΔH_c_* values than those observed in the one-step method.

### 3.4. The Acceleration of MMT on the Crystallization of PTT/PC: Influence of the Mixing Temperature

**Figure 9:**
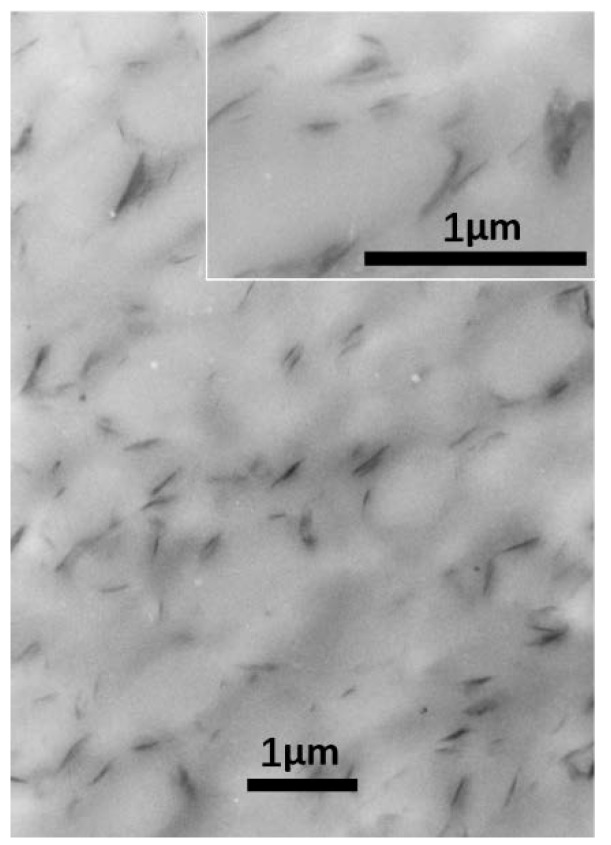
TEM images of the 70/30/1 wt. % PTT/PC/MMT melt processed at 250 °C, 60 rpm, and 10 min. The MMT is Cloisite^®^ 25A.

Comparing the DSC results in Figure 2 and Figure 3, it can be seen that in the presence of MMT and under the same shear rotation speed, the *T_c_* and *ΔH_c_* only change slightly as the mixing temperature is increased from 240 to 250 °C, but they are all lower than the values observed at 240 °C. This is likewise the effect of the increase in shear rate resulting in the increase in crystallization of the PTT/PC without MMT, of which the same shear rate at 240 °C shows a more obvious effect than at the same shear rate at 250 °C [38]. Thus, the greater the inhibition of the crystallization of the PTT phase, the greater the potential for crystallization to be influenced by external factors. Furthermore, compared with the shear rate, the mixing temperature shows a much smaller effect on the MMT promoting the crystallization of the PTT phase; the shear rate significantly influences the dispersion of MMT, and thus, determines the number of induced nuclei. In addition, when the rotation is increased from 60 to 100 rpm at 250 °C, both the *T_c_* and *ΔH_c_* change only very slightly, indicating that the dispersion of MMT is not significantly dependent on the shear rate in the range of our experimental rotations when the temperature approaches 250 °C. This is evidenced by Figure 9, which shows that MMT is almost completely exfoliated, even at 60 rpm.

### 3.5. Effects of the MMT Content on the Crystallization of PTT/PC

**Figure 10:**
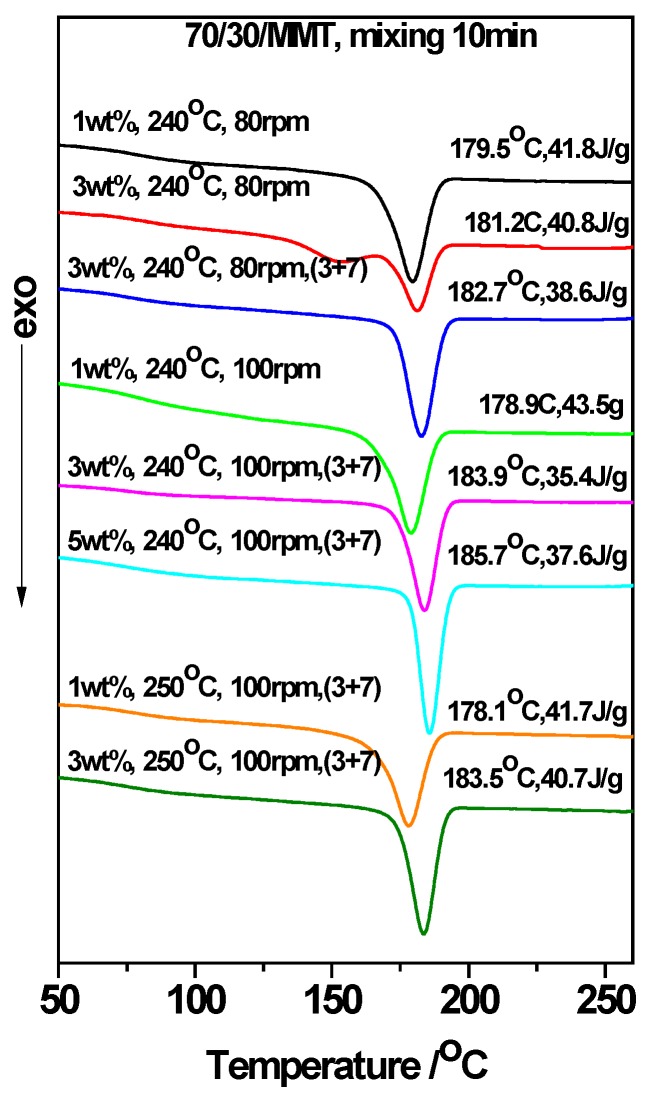
Influences of the MMT (Cloisite^®^ 25A) content on the crystallization of the 70/30/1 wt. % PTT/PC/MMT blend processed at indicated temperatures, shears and processing modes.

Figure 10 shows the effect of the MMT loadings on the crystallization behaviors of 70/30 PTT/PC at different shear rates and via different mixing modes. At 80 rpm and via a one-step mixing method, major and secondary crystallization peaks appear at the peak temperatures of 181.2 °C and 153.5 °C, which are slightly higher and 24 °C lower than the *T_c_* in the presence of 1 wt. % MMT when the MMT content is increased from 1 wt. % to 3 wt. %. Moreover, the overall enthalpy is lower than that of 1 wt. % MMT loading. This multi-peak phenomenon indicates that the promoting effect of MMT on crystallization differs in various areas, causing different crystallization rates of the various PTT segments at different levels, as evidenced in the DSC curve. If the (3 + 7) mode is used at the same shear rate, the secondary crystallization peak disappears, but the peak crystallization temperature and the crystallization enthalpy are raised and decreased slightly, respectively. This shows that the dispersion of MMT is improved, resulting in both an enhanced nucleation effect and enhanced interference with the spherulite growth. The reason for this may be found in the distribution and dispersion of MMT in the matrix. The diffraction patterns of L2 and L3 in Figure 4 shows that the MMT, via two-step mixing, still exhibits a significant diffraction at a low angle, suggesting that the proportion of the intercalated part is considerable. The TEM results in Figure 6 show that the PC dispersed phase in the presence of 3 wt. % MMT (image D) is much finer than that of 1 wt. % (image C). In this case, more MMT is dispersed in the PTT phase, which may be due to the increased MMT loading and the limited interface area not being enough to accommodate the increased MMT content. Consequently, these fine and densely distributed MMT particles, on the one hand, have a strong ability to induce nucleation and therefore increase the peak crystallization temperature, but simultaneously, they also significantly interfere with the growth process and influence the spherulite perfection and crystallinity.

However, the secondary crystallization, when the MMT is at 1–5 wt. % loadings and mixed at 100 rpm, does not appear. This confirms once again that the existence of the secondary crystallization peak is due to the insufficient shear rate of MMT, which leads to insufficient dispersion and thereby the crystallization rate differs in different areas. As the MMT loading is increased from 1 wt. % to 3 wt. %, the *T_c_* increases while the *ΔH_c_* decreases significantly, suggesting that the nucleating ability induced by MMT is increased, but the interference to crystallization growth is also significantly increased. The WAXD results in Figure 4 show that 3 wt. % MMT could not be completely exfoliated at 100 rpm (L3), even though the exfoliation is higher than that at 80 rpm (L2). When the loading is increased from 3 wt. % to 5 wt. %, both the *T_c_* and *ΔH_c_* increase slightly.

### 3.6. The Acceleration of MMT on the Crystallization of PTT/PC: Effect of the Epoxy Resin

**Figure 11:**
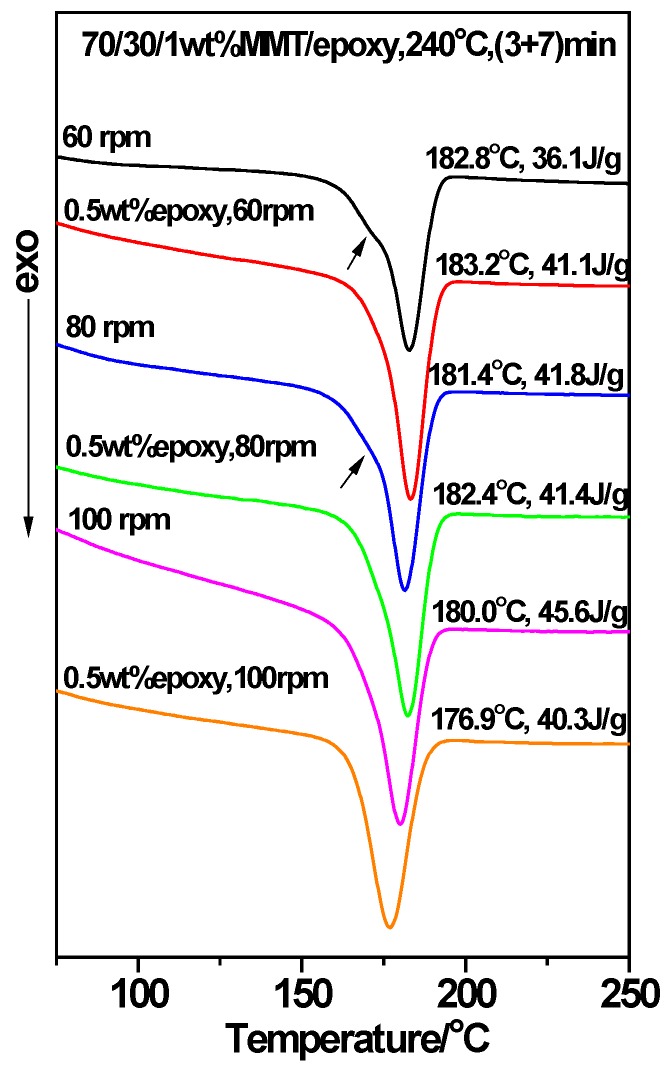
Effects of epoxy on the acceleration of MMT (Cloisite^®^ 25A) on the crystallization the 70/30 PTT/PC blends processed at indicated temperatures and shears.

Considering that the presence of epoxy resin shows a compatibilization effect on the PTT/PC blend, resulting in the phase morphology being changed significantly [27]; therefore, 0.5 wt. % epoxy resin was incorporated into the 70/30/1 wt. % PTT/PC/MMT during processing via the one step method at 60 rpm. Its effect as an interface compatibilizer on the dispersion of MMT was then explored. The DSC results (Figure 11) show that the *ΔH_c_* of the PTT phase is obviously improved, and the secondary crystallization peak disappears (as indicated by the arrow), indicating that the dispersion of MMT is better resulting in enhanced nucleation. The TEM images in Figure 6B also confirm that epoxy resin does improve the exfoliation of MMT. In the case of 80 rpm, the epoxy resin causes the secondary crystallization peak to disappear (as indicated by the arrow), whereas it exhibits little influence on the *T_c_* and *ΔH_c_*, values. However, both the *T_c_* and *ΔH_c_* decrease obviously at a shear rate of 100 rpm. This reveals that the shear rate is not of a sufficient speed to exfoliate MMT completely, and that the incorporation of epoxy resin can promote the exfoliation process and thereby accelerate the crystallization of the PTT phase. Furthermore, the lower the shear strength, the more significant the promotion. When the shear speed is strong enough to completely exfoliate MMT, the addition of epoxy resin leads to a decrease in both the *T_c_* and *ΔH_c_*, suggesting that the crystallization retardation caused by interfacial compatibility is greater than the crystallization acceleration of the PTT phase by MMT.

## 4. Conclusions

MMT shows a significant promoting effect on PTT crystallization. The promoting effect is dependent upon on the distribution and dispersion of MMT, and is related to its loading. Factors such as shear rate, mixing mode, mixing temperature and time, and compatibilizer all influence crystallization. MMT is easier to exfoliate by the two-step method than by the one-step process, and preferentially distributes into the PTT phase or enriches along the phase interface forming an MMT layer that endows the PTT with abundant nuclei; this promotes crystallization much more significantly compared with the one-step method. The increased shear rate leads to an improvement in MMT dispersion and therefore results in an increased nucleation density, and the finer MMT migrates more readily to the interface resulting in a much more smoother phase interface. As the MMT loading is increased, the *T_c_* increases, whereas the *ΔH_c_* decreases, and a secondary crystallization peak appears once the shear is insufficient to finely disperse the MMT. The mixing temperature shows little effect on the acceleration of the crystallization of PTT/PC caused by MMT compared with the shear rate. Only when MMT is not completely exfoliated does the presence of epoxy resin help to promote crystallization because of improved MMT dispersion.

## Supplementary Materials

The following are available online at https://www.mdpi.com/2073-4360/12/3/541/s1, Figure S1: Scheme showing the differences between the surface treatment agents and surface properties of Closite 30B and Closite 25A.
