# Supplementary material for: Prominent Crystallization Promotion Effect of Montmorillonite on PTT/PC Blends with PTT as the Continuous Phase
Source: Polymers (Basel). 2020 Mar 2;12(3):541. doi: 10.3390/polym12030541 (PMC7182810; doi:10.3390/polym12030541)
Supplement: Supplementary file 1 [file polymers-12-00541-s001.pdf]

Supporting Information

Prominent Crystallization Promotion Effect of Montmorillonite on PTT/PC Blends with PTT as the Continuous Phase

Meiling Xue *, Yingjie Liu, Kanghui Lv, Shaowu Han, Shengqiang Gao and Guangshui Yu

Key Laboratory of Rubber-Plastics, Ministry of Education/Shandong Provincial Key Laboratory of Rubber-plastics, Qingdao University of Science & Technology, Qingdao 266042, China; liuyingjie_qust@163.com (Y.L.); 15192569335@163.com (K.L.); hanshaowuqust@163.com (S.H.); gaoshengqiang_qust@163.com (S.G.); yugs@qust.edu.cn (G.Y.)

* Correspondence: meilingxue@qust.edu.cn

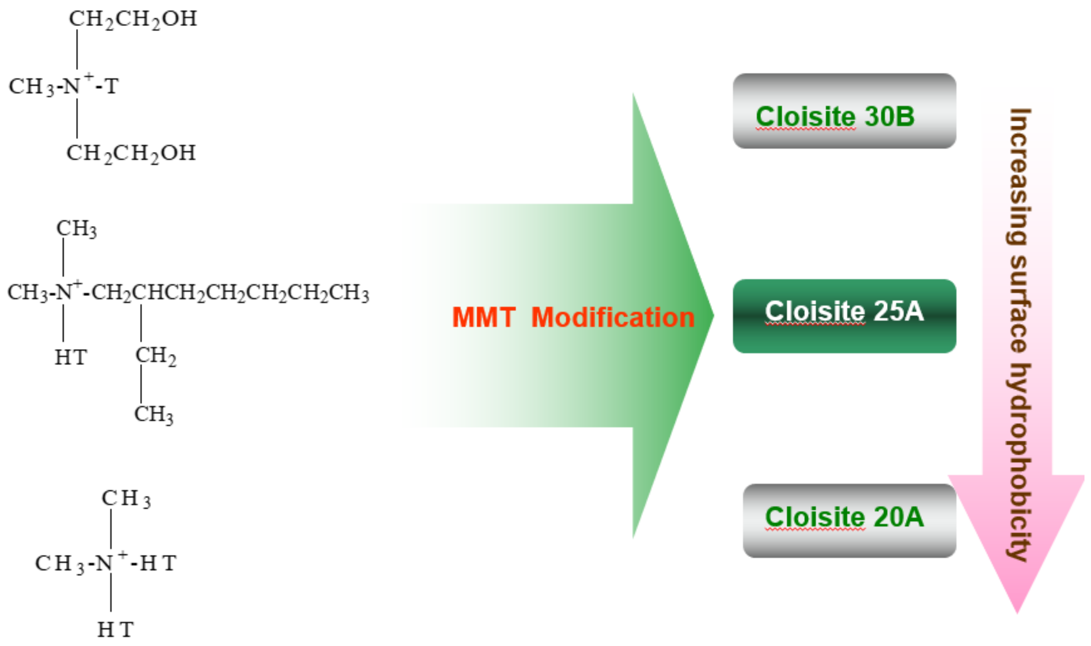

Figure S1. Scheme showing the differences between the surface treatment agents and surface properties of Closite 30B and Closite 25A.
